# Obtaining of Formaldehyde Modified Tars and Road Materials on Their Basis

**DOI:** 10.3390/ma15165693

**Published:** 2022-08-18

**Authors:** Katarzyna Pstrowska, Volodymyr Gunka, Yuriy Prysiazhnyi, Yuriy Demchuk, Yurii Hrynchuk, Iurii Sidun, Marek Kułażyński, Michael Bratychak

**Affiliations:** 1 Department of Advanced Materials Technologies, Wroclaw University of Science and Technology, 7/9 Gdańska Street, 50-344 Wroclaw, Poland; 2 Institute of Chemistry and Chemical Technology, Lviv Polytechnic National University, 12 Bandera Street, 79013 Lviv, Ukraine; 3 Institute of Building and Environmental Engineering, Lviv Polytechnic National University, 12 Bandera Street, 79013 Lviv, Ukraine; 4 Innovation and Implementation Company Ekomotor Ltd., 1A Wyścigowa Street, 53-011 Wroclaw, Poland

**Keywords:** tar, bitumen, formaldehyde, formalin, asphalt concrete

## Abstract

The process of chemical modification of tar and oxidized bitumen with formalin (a 37% aqueous solution of formaldehyde) in a hermetic container was investigated and the effectiveness of the proposed process was proven. It is shown that the most effective raw material for the process is tar, not oxidized bitumen. The expediency and impact of using different types of solvents (toluene, *p*-xylene and petroleum solvent, and *n*-octane) in the modification process were studied. It was established that the solvent should be used in the modification of oxidized bitumens, not tars. The low efficiency of the process of tar modification with formaldehyde without the use of a catalyst was proven, and it was shown that the most active catalyst in the process is sulfuric acid. The influence and optimal values of the main factors controlling the process of chemical modification of tar with formaldehyde were established, namely temperature, duration, and content of the modifier—formaldehyde. On the basis of the found regularities and optimal conditions of the modification process, samples of binding materials (of different brands) with different operational characteristics were obtained, and their comprehensive research was carried out. With the help of FTIR spectroscopy, the chemical interaction of tar with formaldehyde in the presence of an acid catalyst was confirmed. The design of the compositions of asphalt concrete mixtures using formaldehyde-modified tar was carried out, from which cylindrical samples of stone mastic asphalt (SMA-15 brand) were obtained, which were tested according to the main indicators: average density, water-saturation, compression strength at 20 and 50 °C, compression strength after water-saturation (MPa) at 50 °C.

## 1. Introduction

**Figure 1:**
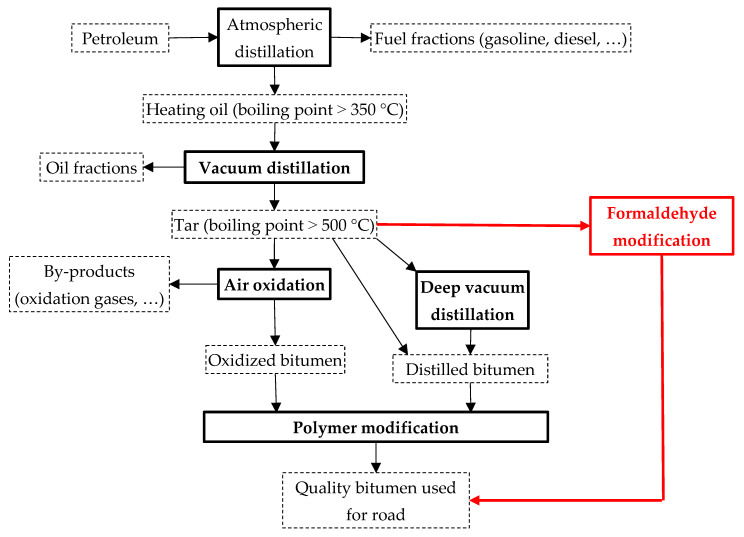
Scheme of bitumen production.

Petroleum bitumen is the main binding material used to produce asphalt concrete mixtures used in road construction and repair. Figure 1 shows a schematic diagram of the production of bituminous materials for road construction (highlighted in black). A number of problems arise when obtaining and applying them. The first of them is the impossibility or difficulty of obtaining high-quality road distilled (residual) bitumen at modern oil refineries, which is explained by the shortage of heavy aromatic oils, which are good raw materials for the production of bituminous materials. The second is insufficient heat resistance and adhesion to mineral materials for distilled and oxidized petroleum bitumens. With an increase in the intensity of traffic, an increase in the volume of heavy goods transportation, the mass of cars, together with the influence of weather and climate factors on the road structure, leads to the destruction of the road surface. The third is to improve the operational properties of distilled and oxidized bitumen; it is necessary to add expensive industrial polymer modifiers to them. As a rule, thermoelastoplasts of the styrene-butadiene-styrene (SBS) type are added to bitumens in the amount of 1–5 wt.% [1,2,3].

Oxidation is by far the best way of chemical conversion of residual crude oil—tar in particular—into road bitumen [4,5,6,7]. This method is economically advantageous because expensive reagents are not needed; oxidation is carried out by air oxygen. The main apparatus used in oxidation plants—oxidation reactor—is structurally quite simple. However, tar oxidation has a number of disadvantages, the main of which is the inability to obtain commercial bitumen with the operational characteristics conforming to existing regulations. As a result, various expensive “physical modifiers” which improve certain parameters [1,2,3,8,9,10] need to be added to oxidized bitumen. Actually, a good way to produce paving bitumen via oxidation is a two-stage method: oxidation of tar and modification of oxidized bitumen with a polymer modifier. In this case, additional modification units and high-cost modifiers are necessary.

Chemical modification of tars and/or bitumens involves changing their chemical structure as a result of the action of the modifier. As a result, products with improved operational characteristics are obtained. Such modification is studied and used on a much smaller scale compared to “physical modifiers” with polymeric materials. It is known from the literature that chemical modification of tar and/or bitumen has been carried out:

- sulfur and high-and low-density polyethylene recycled waste [11], SBS and sulfur [12], the processes combined both physical and chemical modification;
- polyphosphoric acid [13];
- dodecylbenzenesulfonic acid (DBSA), their attachment to asphaltene molecules takes place [14];
- silane coupling agent (SCA) [15];
- thiourea dioxide (ThD) [16];
- maleic anhydride [17,18], maleic anhydride and recycled low-density polyethylene [19].

Bitumen asphaltenes undergo a variety of simple chemical conversions. For example, asphaltenes can be oxidized, sulfonated, sulfomethylated, halogenated, and phosphorylated. The net result is the introduction of functional entities into the asphaltene structure which confer interesting properties on the products for which a variety of uses are proposed [20].

A number of research teams tried to reduce the cost of modifiers by obtaining them from relatively cheap raw materials. For example, several types of paving bitumen modifiers of physical action were synthesized from relatively cheap by-products of coking coal [21,22,23,24,25,26] at the Department of Oil and Gas Processing of Lviv Polytechnic National University. However, industrial-scale production of the modifiers so developed does not exist.

In this situation, there is a need to find alternative production methods of paving bitumen from tar without the modifiers. We report below a way to produce paving bitumen via modifying tar (residue from oil refining) with a relatively cheap low-molecular weight organic substance—formaldehyde. This is a chemical modification—which in the case of its industrial implementation will avoid the use of expensive modifiers (Figure 1; highlighted in red). Importantly, the proposed method can be carried out using the existing oxidation reactors—without their conversion or with only minor design changes.

## 2. Materials and Methods

### 2.1. Characteristics of Initial Materials

Tar and two samples of oxidized bitumen (JSC Ukrtatnafta, Kremenchuk, Ukraine) were used as raw materials for experimental research, namely: tar (T1); oxidized bitumen (OB1) of BND 60/90 brand; oxidized bitumen (OB2) of BND 70/100 brand. Their main characteristics are given in Table 1.

Formalin, a 37% aqueous formaldehyde solution, was used for the chemical modification of tar and bitumen.

The following substances were used as the process catalysts: concentrated (37%) hydrochloric acid (HCl), analytical grade, density 1.19 g/cm^3^; concentrated sulfuric acid (H_2_SO_4_); density 1.83 g/cm^3^; sodium hydroxide (NaOH), hygroscopic crystals, density 2.13 g/cm^3^.

### 2.2. Synthesis of FMT and FMOB

The products obtained after modification have qualitative characteristics allowing them to be classified as commercial bituminous road materials that meet the requirements of the standards.

**Figure 2:**
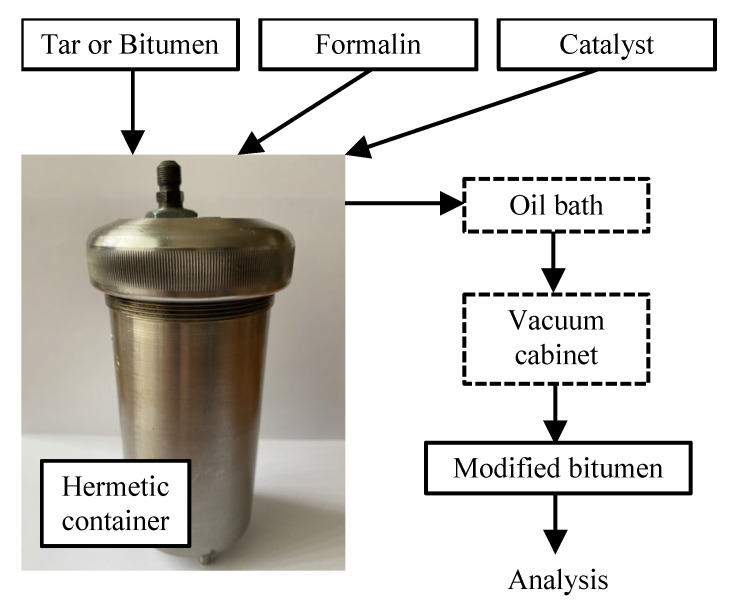
Scheme of the stages of work on the modification of tar and bitumen with formaldehyde.

Chemical modification with formaldehyde without stirring was performed in hermetic containers capable of operating under high pressure (Figure 2). This is due to the ability of the modifier to evaporate at the operating temperatures (60–160 °C). At the loading stage, the temperature in the container must be lower than the boiling point of the modifier.

The scheme of the stages of work on the modification of tar and bitumen with formaldehyde is presented in Figure 2. The container is loaded with raw materials (tar or bitumen) and heated to a temperature that ensures that it is fluid, after which, the raw material is allowed to be cooled to a temperature below the boiling point of formalin. Next, a pre-prepared mixture is added: a chemical reagent (formalin) and a catalyst in the required ratio. The container is sealed, placed in an oil bath at the required temperature and the start time is recorded. After completion of the modification process, the depressurized container is placed in a vacuum cabinet to remove water and unreacted components. Vacuum drying is performed for 4 h at 120 °C.

Before studying the effect of mixing, the modification process was performed in a hermetic container with mixing.

### 2.3. Raw Material and Products Analyses

#### 2.3.1. Bitumen Analyses

Parameters were determined according to the standards: penetration at 25 °C EN 1426, softening point EN 1427, ductility at 25 °C [27], Fraass breaking point EN 12593, penetration index EN 12591, and RTFOT method EN 12607-1.

Bitumen adhesion to stone material was determined according to [25,28]; rolling bottle tests were conducted according to EN 12697-11. We used crushed stone of 8/11 and 20/40 mm fractions obtained from natural stone (S1) and selected at the LLC Novograd-Volyn Stone Crushing Plant (Zhengzhou, China).

Fourier-transform infrared (FTIR) spectra were recorded on a Thermo scientific Nicolet iS10 (Waltham, MA, USA) interference Fourier spectrophotometer (Thermo Fisher Scientific, Waltham, MA, USA). The FTIR spectra were investigated in the transmission mode within the wavenumber range of 4000–600 cm^−1^ with the spectral resolution set to 4 cm^−1^. The IR spectrophotometer was equipped with a diamond Attenuated Total Reflectance (ATR) unit with a spectral range cutoff of 525 cm^−1^. The electronic spectrogram obtained in electronic format was evaluated on a computer using the software for that spectrophotometer.

#### 2.3.2. Preparation and Testing of SMA-15

Crushed stone (10/15 and 5/10 mm) and crushed stone screening (0.071/5 mm) from JSC Gayvoronsky Speckarier (Hayvoron, Ukraine) and mineral powder from LLC Skala-Podilsky Specialized (Skala-Podilsky, Ukraine) career were used to make SMA-15 samples.

The grain composition of the mineral powder is given in Table 2.

To prevent bitumen bleeding, a Celbit stabilizer consisting of 80 wt. % cellulose and 20 wt.% bitumen was used. Celbit meets the requirements for cellulose fiber [29].

Stone mastic asphalts (SMAs) were studied according to a Ukrainian procedure [30] in the form of cylindrical samples (diameter and height 71.4 mm; weight 655.0 g). The average density of each SMA was determined by hydrostatic weighing. Residual porosity was determined by SMA pore volume based on pre-set average density of cylindrical samples and the actual density of the SMA mix. Water-saturation was determined by the amount of water absorbed by a sample at a pre-set mode of saturation in a vacuum unit. The compression strength at 20 and 50 °C was determined on mechanical presses with a press-plate movement speed of 3.0 ± 0.1 mm/min. For testing, SMA samples are installed under the press plates with flat upper and lower faces of the cylinder. Before testing, the samples are thermostat-conditioned in a vessel with water for 60 ± 5 min at the temperatures of 50 ± 1 °C and 20 ± 1 °C. The samples for testing compression strength at 50 °C are placed (before the thermostat-conditioning) into tight polyethylene bags—to prevent their contact with water. Compression strength after water-saturation was determined according to the above methods for samples that were tested for water-saturation.

## 3. Results and Discussion

**Figure 3:**
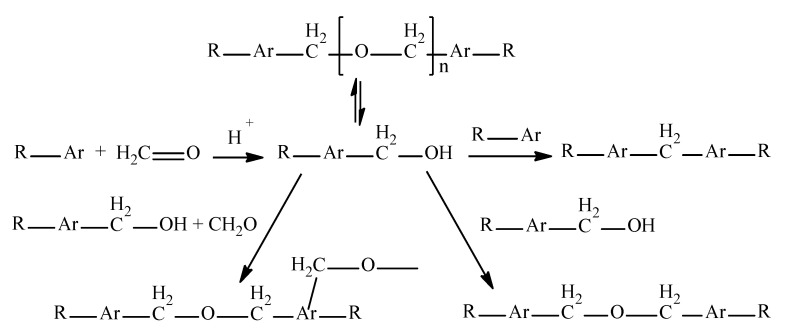
Chemistry of aromatic compounds copolycondensation with formaldehyde.

It is known that individual arenes and asphaltene resin substances (ARSs, that is oils, resins, and asphaltenes) are able to enter into a copolycondensation reaction with formaldehyde to form arene-formaldehyde resins and formalites, respectively [31,32]. According to these reports, the copolycondensation of aromatic fragments is a complex set of reactions resulting in the formation of methylol, ether, acetal, and methylene groups (Figure 3).

Thus, the following transformations occur during the modification process:

Oils → Resins → Asphaltenes

The degree of polycondensation of such copolymers is not high due to the rapid gel effect caused by the significant size of the ARS molecules [31].

We infer that compounds with benzene rings—which are present in petroleum residues—may react with formaldehyde and thus improve the service properties of the tar. We expect that this will improve the properties of paving bitumen as well.

The first stage of the project was the investigation of the influence of the process’s main parameters and conditions, namely the effect of mixing, solvent addition, modifier (formalin) amount, nature and amount of the catalyst, process temperature, and duration.

### 3.1. Influence of Process Conditions and Parameters

#### 3.1.1. Effect of Mixing

**Figure 4:**
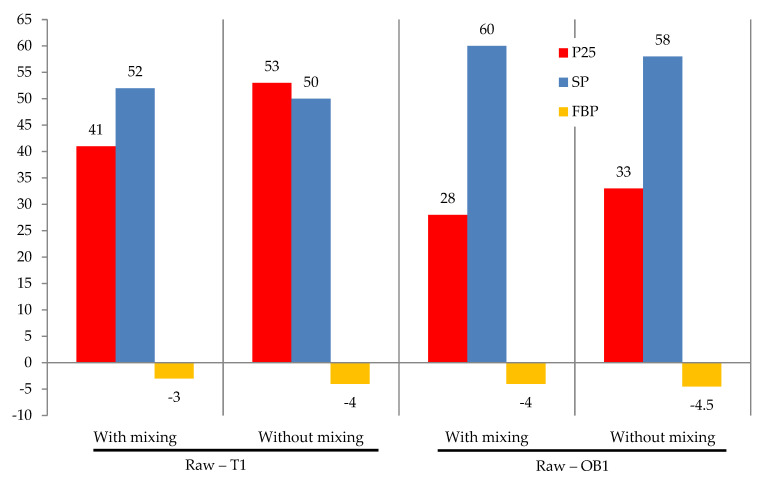
The effect of mixing on the modification process: toluene amount was 40.0 kg/100 kg raw; formalin amount (including formaldehyde) 10.0 (3.7) kg/100 kg raw; catalyst amount (HCl) 2.5 kg/100 kg raw; temperature 120 °C; duration 3 h.

Since the studies on the modification of oil residues with formaldehyde must be performed in a hermetic container due to the high volatility of formaldehyde, it was first necessary to find out how mixing affects the process. Thus, four experiments were performed for T1 and OB1 (two experiments with constant stirring and two—without stirring). The experimental conditions and some characteristics of the products so obtained are provided in Figure 4. The following important parameters were determined: penetration at 25 °C; softening point (SP); and Fraass breaking point (FBP).

The initial parameters of petroleum residue modification, namely the content of solvent (toluene) and formalin, the nature and amount of the catalyst, modification temperature, and duration were chosen based on the data presented by Moshchinskaya [31].

It is clear from Figure 4 that the mixing had a minor effect on the efficiency of the chemical modification of oil residues with formaldehyde. This could be due to the fact that the ARSs reaction with formaldehyde proceeds at a high rate; the methylol carbo-cation, according to Equation (1), is a highly reactive process and rapidly interacts with ARSs (Equation (2)), as reported by Moshchinskaya [31].

(1) $$H_{2}C=O+H^{+}\to H_{2}C^{+}\;–\;OH$$

(2) $${\mathrm{ARS}}_{S}+H_{2}C^{+}\;–\;OH\underset{–H^{+}}{\to }{\mathrm{ARS}}_{S}\;–\;{\mathrm{CH}}_{2}\;–\;\mathrm{OH}$$

#### 3.1.2. Effect of Solvent

It is known that individual aromatic hydrocarbons (benzene, toluene, xylenes, and others) are able to enter into the polycondensation reaction with formaldehyde—as reported by [33,34]. When modifying oil residues with formaldehyde, two kinds of solvents were used to reduce the viscosity of the reaction mixture. The first type is active in polycondensation reactions with formaldehyde (toluene, *p*-xylene, and petroleum solvent), and the second one (*n*-octane) is passive.

**Figure 5:**
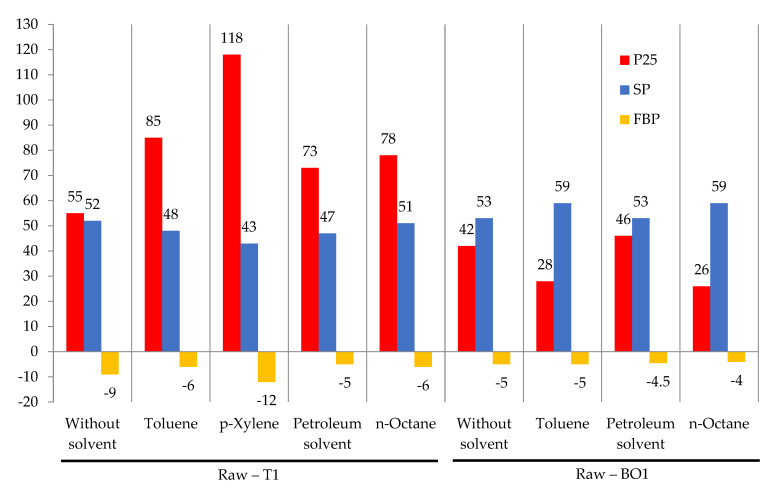
The effect of solvent on the modification process; solvent amount 20.0 kg/100 kg raw; formalin amount (including formaldehyde) 10.0 (3.7) kg/100 kg raw; catalyst amount (HCl) 2.5 kg/100 kg raw; temperature 120 °C; duration 3 h.

Process conditions and characteristics of the obtained products are shown in Figure 5.

In our opinion, it is advisable to use a solvent/diluent when modifying a more viscous and condensed petroleum residue—oxidized bitumen (OB1) due to the greater gel effect (Figure 5). When modifying oxidized bitumen (OB1) without solvent, the softening point increased by 7 °C, and when using toluene, petroleum solvent, and *n*-octane, the softening point increased by 13, 7, and 13 °C, respectively. When modifying solvent-free tar (T1), the softening point increased by 13 °C, and when using toluene, *p*-xylene, petroleum solvent, and *n*-octane—by 9, 4, 8, and 12 °C, respectively. That is, the addition of solvent is effective only when modifying the more viscous compound (OB1). When modifying tar (T1), the solvent is not advisable to use. Therefore, subsequent studies on the modification process were performed using solvent-free tar (T1).

#### 3.1.3. Effect of Catalyst Nature and Amount

At first, it is necessary to determine which type of catalyst is most appropriate in the process of modifying tar with formaldehyde. It is known from the literature [31] that various acids and alkalis can be effective catalysts for the polycondensation of arenes with formaldehyde (production of arene-formaldehyde resins). To verify this, a concentrated hydrochloric acid, sulfuric acid, and dry sodium hydroxide were used.

**Figure 6:**
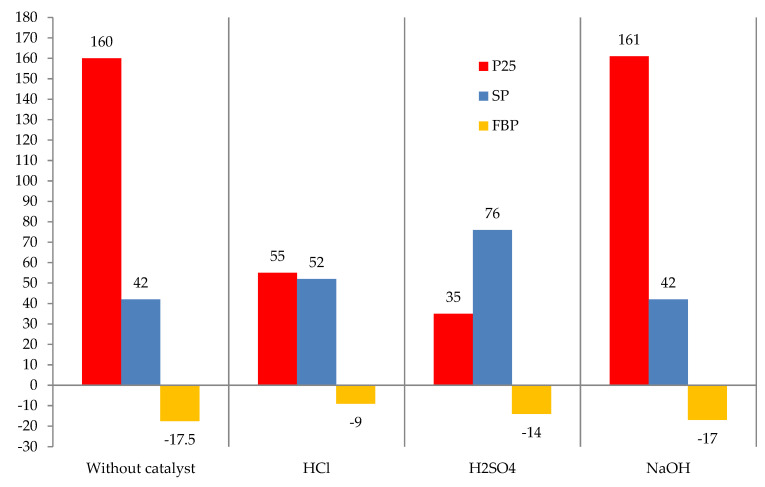
The effect of catalyst nature on the modification process: raw is T1; formalin amount (including formaldehyde) 10.0 (3.7) kg/100 kg raw; catalyst amount HCl or H_2_SO_4_ = 2.5, NaOH = 1.0 kg/100 kg raw; temperature 120 °C; duration 3 h.

The effect of the catalyst nature on the tar modification with formaldehyde is shown in Figure 6.

Figure 6 shows that the most effective catalysts for the reaction of modification of formaldehyde tar are acids. Sulfuric acid was much more active than hydrochloric acid, because the obtained modified tar has a much higher softening point and a lower value of the Fraass breaking point, i.e., it is characterized by a wider plasticity interval. Thus, further studies were carried out using sulfuric acid as the catalyst.

**Figure 7:**
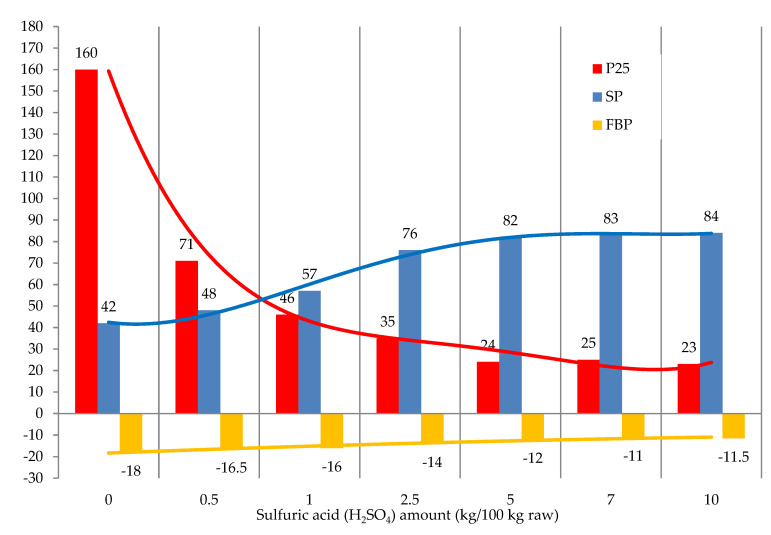
The effect of catalyst amount (H_2_SO_4_) on the modification process: raw is T1; formalin amount (including formaldehyde) 10.0 (3.7) kg/100 kg raw; temperature 120 °C; duration 3 h.

The effect of the sulfuric acid amount on the operating properties of modified bitumen is shown in Figure 7.

The amount of sulfuric acid over 2.5 kg/100 kg raw had virtually no effect on the softening point and penetration of the resulting products. At and above the catalyst amount of 7.0 kg/100 kg raw, the resulting products became heterogeneous, and clots formed. We infer that the best characteristics of the modified tar can be achieved with 2.5 kg/100 kg raw of sulfuric acid (Figure 7).

#### 3.1.4. Effect of the Temperature on the Modification Process

**Figure 8:**
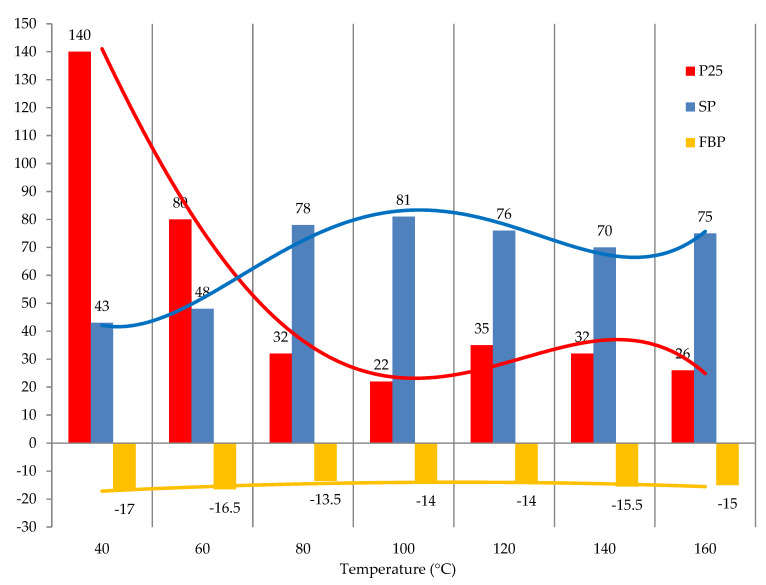
The effect of temperature on the modification process: raw is T1; formalin amount (including formaldehyde) is 10.0 (3.7) kg/100 kg raw; catalyst amount (H_2_SO_4_) is 2.5 kg/100 kg raw; duration 3 h.

To study the effect of temperature on the process of modifying tar with formaldehyde in the presence of acid catalyst, the results from the literature [31] regarding the condensation reaction of arenes with formaldehyde were used. The polycondensation temperature of arenes (benzene, toluene, xylenes, naphthalene, anthracene, and others) with formaldehyde was in the range from 40 to 60 °C. At the same time, it is necessary to take into account the high viscosity of the original tar in comparison with low molecular arenes. Therefore, studies on the temperature effect were carried out at higher temperatures, in particular in the range from 40 to 160 °C. The results obtained are shown in Figure 8.

One can see that the temperature below 100 °C significantly affects the characteristics of the obtained products. Further increase in temperature to 120 °C and above has a minor effect on the characteristics of the modified tars and even leads to a slight decrease in the softening point (Figure 8). Thus, it can be concluded that the most favorable temperature for modifying tar with formaldehyde in the presence of sulfuric acid is 80–120 °C.

#### 3.1.5. Effect of the Modification Duration

**Figure 9:**
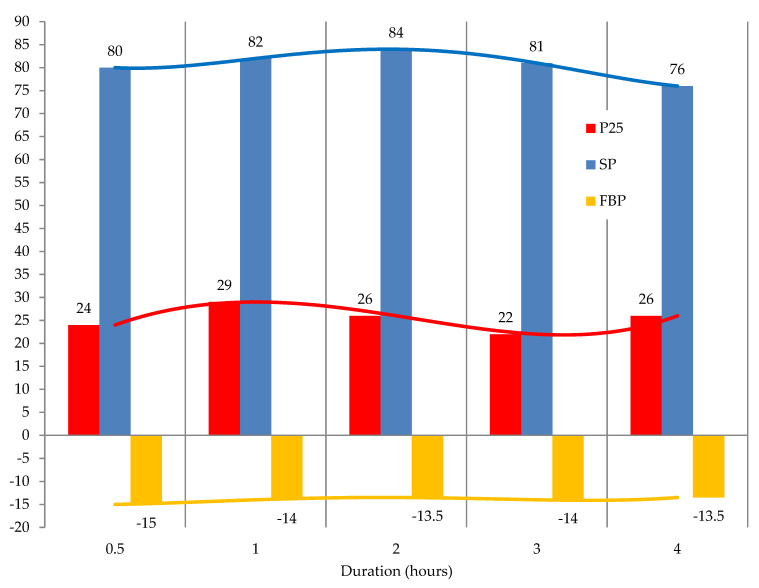
The effect of time on the modification process: raw is T1; formalin amount (including formaldehyde) is 10.0 (3.7) kg/100 kg raw; catalyst amount (H_2_SO_4_) is 2.5 kg/100 kg raw; temperature 100 °C.

The effect of duration on the modification process is shown in Figure 9.

In our opinion, formalin itself should not be regarded as the modifier of this process; the modifier is a mixture of formalin and acid in a certain ratio (formulating agent). Formalin and acid interact with each other (Equation (1)) and form a highly reactive methylol carbo-cation. The addition of this highly reactive carbo-cation (^+^CH_2_–OH) to arene (Equation (1)) occurs very rapidly during the first 0.5 h. Some of the subsequent transformations, shown in Figure 3, also occur during vacuum drying.

The results shown in Figure 9 indicate that the main transformations of the original tar into modified tar in the presence of H_2_SO_4_ occur for 0.5 h. Further increase in the process duration does not lead to significant changes in the characteristics of the products.

#### 3.1.6. Effect of Formaldehyde Amount on the Modification Process

**Figure 10:**
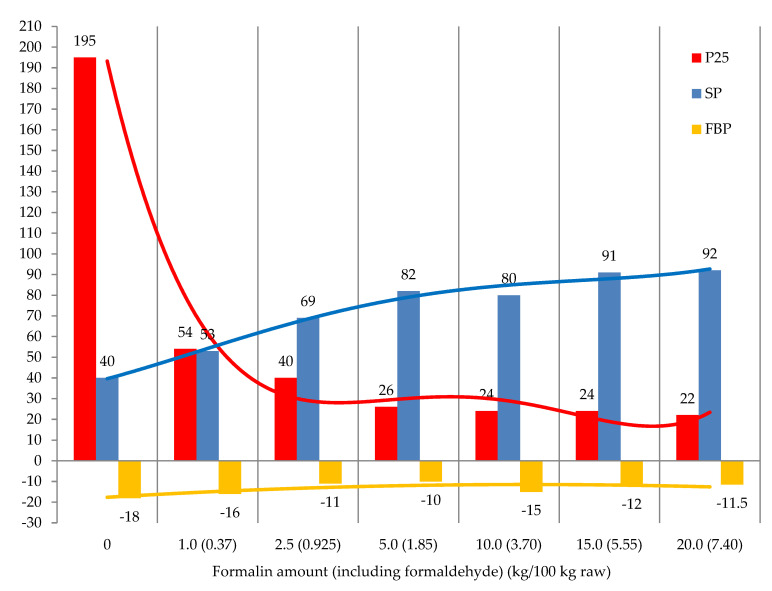
The effect of formalin amount on the modification process: raw is T1; catalyst amount (H_2_SO_4_) is 2.5 kg/100 kg raw; temperature 100 °C; duration 0.5 h.

The next stage of the investigation was to establish how the amount of formaldehyde affects the characteristics of the resulting products under the above-mentioned conditions. For comparison, we studied the modification of tar in the absence of formaldehyde. The experimental results are represented in Figure 10.

The increase in formalin (formaldehyde) amount to 2.5–5.0 kg/100 kg raw at the same amount of the catalyst (concentrated sulfuric acid) increases softening point to 82 °C and decreases penetration to 26. A further increase in the formalin amount had almost no effect on SP and P25. Therefore, the optimal amount of formalin (at the amount of H_2_SO_4_ 2.5 kg/100 kg raw) was 2.5–5.0 kg/100 kg raw, which corresponds to a formaldehyde amount of 0.925–1.850 kg/100 kg raw, and a formalin/catalyst ratio of 1–2.

**Figure 11:**
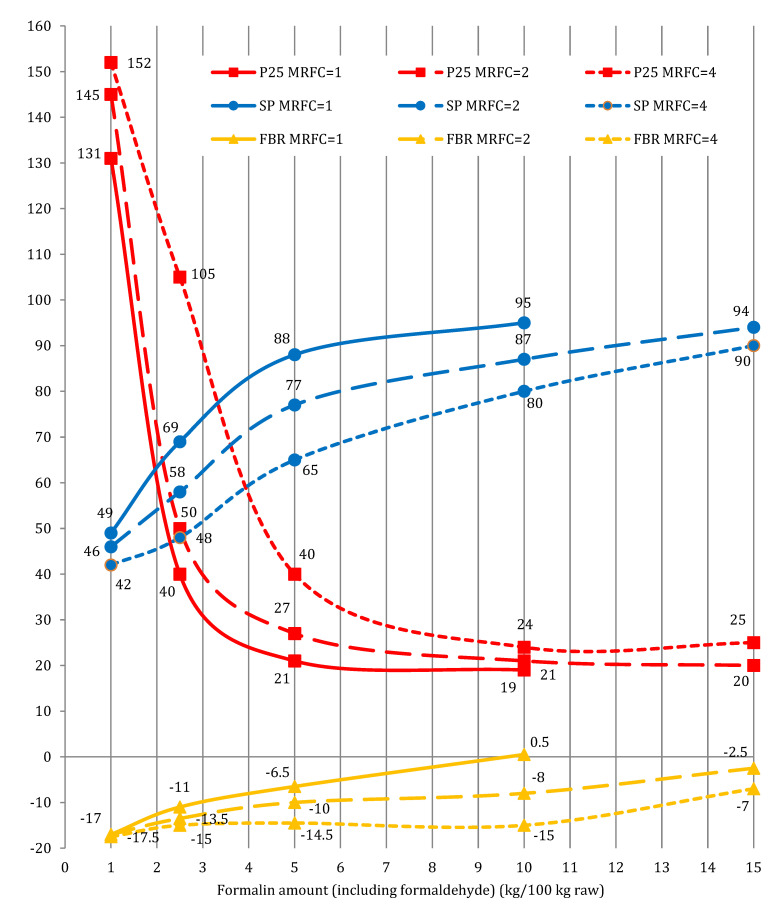
The effect of MRFC on the modification process: raw is T1; catalyst H_2_SO_4_; temperature 100 °C; duration 0.5 h.

As was mentioned above, formulating agent (a mixture of formalin and concentrated sulfuric acid in a certain ratio) should be regarded as the process modifier. Thus, the determination of the optimum mass ratio formalin/catalyst (MRFC) was the next stage of investigations. Three values of MRFC (1, 2, and 4) were studied in the range of formalin amounts from 1.0 to 15.0 kg/100 kg raw. The results are presented in Figure 11.

At the same amount of formalin, the increase in MRFC from 1 to 4 decreases the value of SP and increases P25. That is, it is more expedient to use a formulating agent (modifier) with MRFC equal to 1.0. At the same value of MRFC, the increase in formalin amount from 1.0 to 10.0 kg/100 kg raw increases SP of the obtained bitumen, and decreases its P25 (Figure 11). Thus, the optimum amount of formalin in the process of tar chemical modification with formaldehyde was 5 wt.% and MRFC was 1.

### 3.2. Conditions for Obtaining FMT

With the help of mathematical treatment of the results, we selected the process conditions under which it is possible to obtain binding materials which meet the requirements of the Ukrainian standards regarding the heat resistance (SP) for the following grades of bitumen:

- BND 70/100 (DSTU 2019b)—FMT-1 (using sulfuric acid as the catalyst) and FMT-4 (using hydrochloric acid as the catalyst);
- BMPA 70/100-55 (DSTU 2021)—FMT-2;
- BMPP 35/50-70 (DSTU 2021)—FMT-3.

A more detailed mathematical treatment of the results of the study of the tar modification process with formaldehyde is presented in [35].

The conditions for obtaining FMT are provided in Table 3.

### 3.3. FTIR Study of FMT

**Figure 12:**
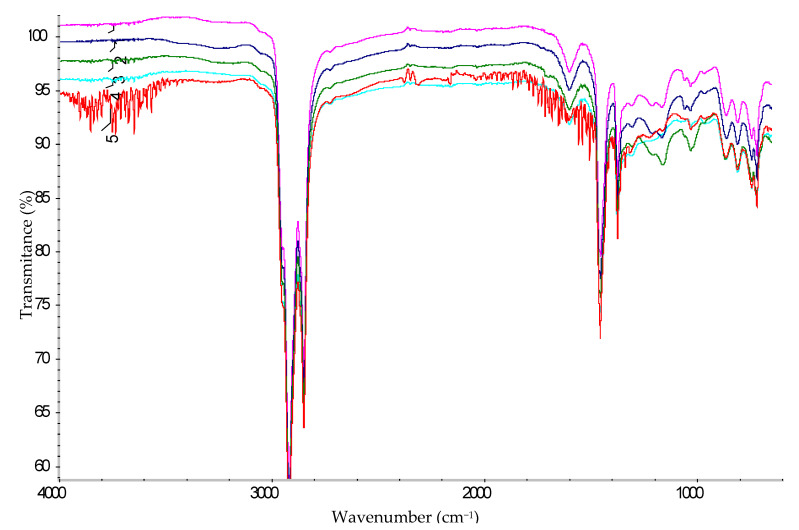
FTIR spectrum (4000–600 cm^−1^): 1—FTM-1; 2—FTM-2; 3—FTM-3; 4—FTM-4; 5—T1.

**Figure 13:**
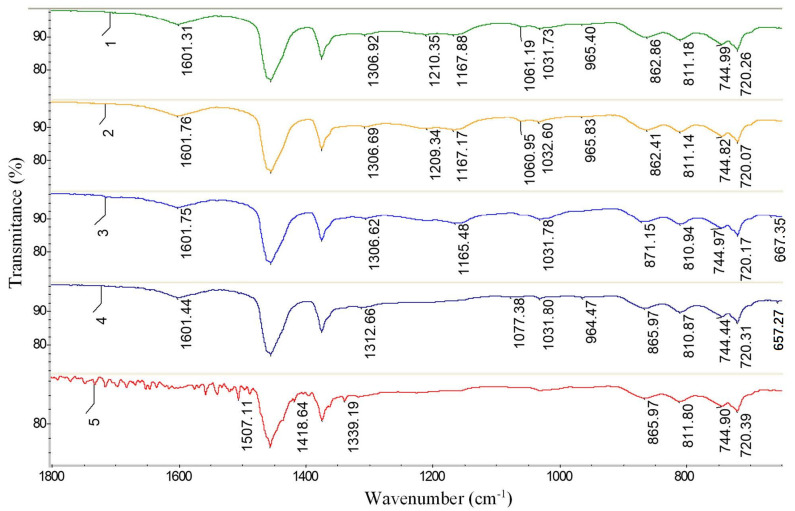
FTIR spectrum (1800–650 cm^−1^): 1—FTM-1; 2—FTM-2; 3—FTM-3; 4—FTM-4; 5—T1.

To confirm the structure of the obtained FMT, FTIR analysis was used. For comparison, the FTIR spectra of FTM-1, FTM-2, FTM-3, FTM-4, and T1 were analyzed (Figure 12 and Figure 13).

As was mentioned above, the FTIR spectrum of the original tar (Figure 12 and Figure 13) contains an absorption band at 1457 cm^−1^, which corresponds to the stretching vibrations of the benzene ring. Benzene rings are disubstituted molecules, as indicated by the absorption bands at 812 and 744 cm^−1^, and trisubstituted ones (absorption band at 720 cm^−1^), which are part of the tar structure and interconnected by methylene bonds. This is confirmed by absorption bands at 2919 and 2850 cm^−1^. In addition, the molecules of the original tar contain free methyl groups, which are proved by symmetrical deformation vibrations at 1375 cm^−1^, characteristic of the –RCH(CH_3_)_2_ fragment.

Benzene rings were also detected in the FTIR spectra of FMT (Figure 12 and Figure 13), which were confirmed by the absorption bands at 1601 and 1456 cm^−1^. The presence of –CH_2_– groups is proven by absorption bands at 2919 and 2850 cm^−1^. Absorption bands at 863–862, 811 and 745 cm^−1^ in the modified tar indicate the presence of substituted benzene rings in the products.

Absorption bands at 1376–1375 and 1061 cm^−1^, which may indicate the presence of –CH_2_O– groups and the ester bond –C–O–C–, respectively [36,37], were found in the spectra of modified tars, in contrast to the spectrum of the original tar (Figure 12 and Figure 13). This fact confirms the chemistry of the modification process shown in Figure 3, i.e., the entry of formaldehyde molecules into the tar structure. Moreover, in the FMT-1, FMT-2, and FMT-3 spectra (Figure 12 and Figure 13), the absorption bands at 1210–1209, 1167, and 1033–1032 cm^−1^ were found; they can be attributed to the vibrations of the –RSO_3_H fragments [36,37]. These bands were absent in T1 and FMT-4 spectra, indicating the dual role of sulfuric acid in the process of tar modification. On the one hand, H_2_SO_4_ acts as a catalyst for the reaction of formaldehyde with aromatic compounds of tar, and, on the other hand, it reacts with the tar components.

### 3.4. FMT Performance

The physical and mechanical properties of FMTs are given in Table 4.

The properties of FMT-1 and FMT-4 samples were compared with the requirements for oxidized petroleum bitumen of BND 70/100 grade, because this grade is the most widely used in road construction. FMT-2 and FMT-3 were compared with polymer-modified bitumen of BMPA 70/100-55 and BMPP 35/50-70 grades, respectively (Table 4).

One can see from Table 4 that the raw material—tar (T1)—does not meet the requirements for bitumen BND 70/100. Formaldehyde-modified tars (FMT-1 and FMT-4) meet the requirements, except for indices P25 and D25. A value of P25 above the requirements is an advantage; this indicates the high plasticity of bitumen. Thus, such bitumen will be a good raw material for its modification with traditional commercial polymers such as styrene-butadiene-styrene (SBS), styrene-isoprene-styrene (SIS), and others. This is an object of further investigation.

Bitumen FMT-2 and FMT-3 meet all requirements for polymer-modified bitumen of BMPA 70/100-55 and BMPP 35/50-70 grades, respectively (Table 4). The exception is the value of E25. It should also be noted that FMT-2 and FMT-3 have significantly higher SP than the normative values. This will reduce the amount of the traditional polymer (such as SBS elastomer) needed for the E25 to achieve the required value. This is also an object of further investigation.

**Figure 14:**
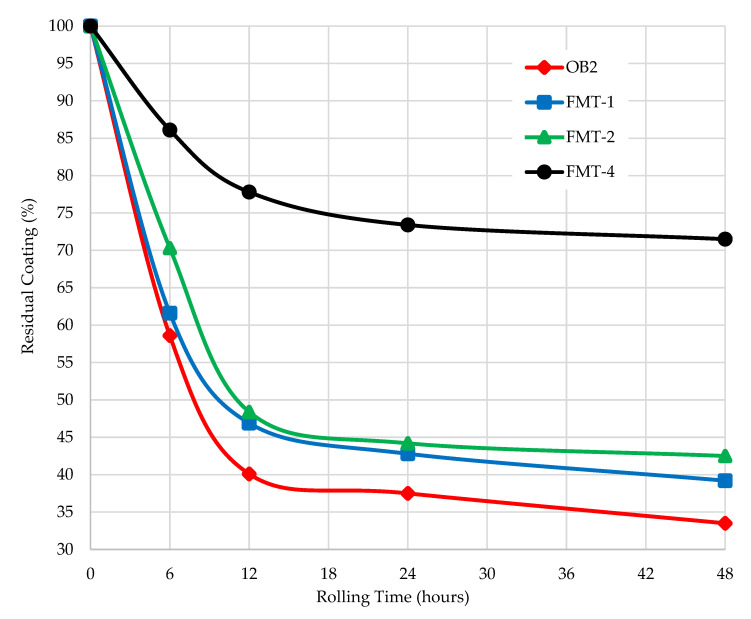
Results of the rolling bottle tests with S1.

**Figure 15:**
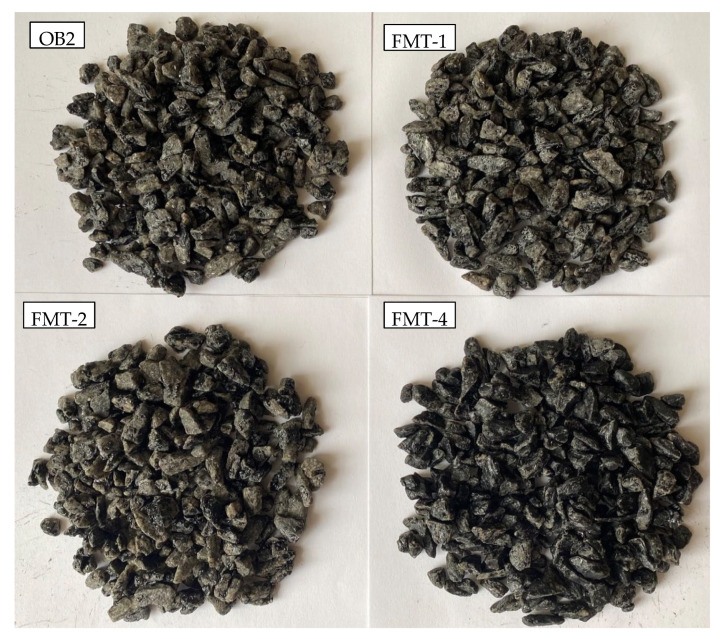
Visual estimation of aggregate surface area covered with bitumen after 48 h.

The adhesion of FMT-1, FMT-2, and FMT-4 to traditional acid fillers of asphalt concrete (S1) were studied by the Rolling Bottle Test method (Figure 14 and Figure 15).

The results show that the degree of coverage for the samples FMT-1, FMT-2, and FMT-4 was higher compared to that for the original bitumen. During the first 12 h, an intensive decrease in the coverage rate is observed, then this process slows down and the rate was not changed during the following 36 h. After 48 h of testing, the degree of coverage for FMT-1, FMT-2, and FMT-4 was 39.2%, 42.5%, and 71.5%, respectively, and the degree of coverage for original bitumen (OB2) was 33.5% (Figure 14). This indicates a lower tendency of FMT-1, FMT-2, and FMT-4 samples to peel off; they also had a higher bond strength of the binder to the aggregate (crushed stone) compared to the original sample (OB2).

It should be noted that the degree of coverage for FMT-4, which was obtained using concentrated hydrochloric acid as the catalyst, is much higher than for other samples (FMT-1 and FMT-2), which were obtained using concentrated sulfuric acid. In particular, after 6 h of testing, the degree of crushed stone coverage for FMT-4 is 86.1%, while for the other samples, this value is much lower, 58–70%. This is due to the fact that in the process of FMT vacuum drying after modification, hydrochloric acid is removed from it, but sulfuric acid remains due to its higher boiling point. Sulfuric acid provides FMT with high acidity, and on contact with acidic fillers (S1) these samples peel off (acid bitumen and acid crushed stone repel each other). This property of FMT obtained using concentrated sulfuric acid will be taken into account in further studies.

### 3.5. Formation and Testing of Asphalt Concrete

The selected composition of SMAM-15 is given in Table 5. It is a basic composition with different binders (OB2, FMT-1, FMT-2, FMT-3, and FMT-4).

The binder amount in SMAM-15 was constant. The manufacturing and compaction temperatures of SMAM-15 comply with those required by standards (DSTU 2015b and 2016).

The physical and mechanical properties of compacted samples of SMA-15 using different binder variants are given in Table 6. The binder bleeding from SMAM-15 for all samples was not more than 0.20 wt.% according to DSTU (2015b). Requirements for physical and technical parameters of SMA-15 were regulated by DSTU (2015b). Shift 1 was for the climate region A-2, which includes the city of Lviv.

When analyzing Table 6, we observe that all SMA-15 with FMT meet regulatory requirements. The comparison of different FMT showed the best results for FMT-4—obtained using hydrochloric acid as the catalyst. The compression strength at 20 and 50 °C for this sample (2.6 and 1.1 MPa) was higher compared with other FMT (1.5–2.2 and 0.6–1.0 MPa).

Stone mastic asphalt (SMA-15) with acidic FMT-1 (T1 modified in the presence of H_2_SO_4_) was characterized by a low value of compression strength at 50 °C (0.6 MPa) compared to SMA-15 using a traditional binder OB2 (1.7 MPa). With the increasing softening point of FMT (in the series FMT-1 → FMT-2 → FMT-3), the value of compression strength at 50 °C increases to 1.0 MPa, but this is not enough. SMA-15 with FMT-4 (T1 modified in the presence of HCl) was characterized by higher values of compression strength at 50 °C (1.1 MPa) compared to FMT obtained using the catalyst H_2_SO_4_ (FMT-1, FMT- 2 and FMT-3). Low values of compression strength are caused by poor adhesion between the acidic binder and acidic filler—confirmed by the rolling bottle test (Figure 12).

From Table 6, it is also seen that after water saturation of the SMA-15 samples, the value of compression strength is practically not changed for OB2 and FMT-4 (OB2 1.7 → 1.8; FMT-4 1.1 → 1.1). In contrast, this value increases more than twice for SMA-15 with FMT-1, FMT-2, and FMT-3.

Thus, the quality of SMA obtained using FMT depends on the conditions of its production, mainly the nature of the catalyst used.

## 4. Conclusions

The influence of process parameters on bitumen production via modification of oil residue (tar) with formaldehyde has been established. The addition of a solvent to reduce the viscosity of the reaction mixture is effective only for the modification of more viscous oxidized bitumen, while it is not advisable for the modification of tar. Sulfuric acid, hydrochloric acid, and crystalline sodium hydroxide were studied as the catalysts for the process of tar chemical modification with formaldehyde in the form of a 37% aqueous solution of formalin. Sulfuric acid in the amount of 2.5 kg/100 kg raw was found to be the most effective catalyst.

The amount of formaldehyde 0.925–1.85 kg/100 kg raw can be considered an optimum amount, which corresponds to the formalin / H_2_SO_4_ ratio = 1 (*w*/*w*) at the temperature of 100 °C and duration of 0.5–1.0 h. The entry of molecules into the structure of the modified tar is confirmed by absorption bands at 1376–1375 cm^−1^ and 1061 cm^−1^, which indicate the presence of the –CH_2_O– group and the ether bond –C–O–C–, respectively. Based on the determined physical and mechanical properties of the modified tars, it was found that due to chemical modification, the thermal performance of products corresponding to commercial paving bitumen was improved.

It was shown that the production of road binding materials via tar chemical modification with formaldehyde is a quite flexible process since it allows to produce bitumen with a softening point from 47.4 to 83.4 °C (i.e., with different viscosity values), depending on the selected parameters of the process.

The adhesion properties, determined by the rolling bottle test, were much better for the samples modified in the presence of concentrated hydrochloric acid than for the samples obtained using concentrated sulfuric acid. Stone mastic asphalt samples were prepared using bitumen (formaldehyde modified tars) and tested. All the obtained samples of asphalt concrete meet the Ukrainian standard requirements for the SMA-15 grade. Asphalt concrete based on bitumen obtained by tar modification with formaldehyde in the presence of hydrochloric acid is characterized by the best parameters.

## Figures and Tables

**Table 1:** Physico–chemical characteristics of raw materials.

Index	Material			Method
	T1	OB1	OB2	
Density at 20 °C (kg/m^3^)	982.9	–	–	EN 15326
Initial boiling point (°C)	386	–	–	–
Flashpoint (°C)	282	–	–	EN ISO 2592
Penetration at 25 °C (0.1 mm)	247	71	82	EN 1426
Softening point (°C)	39.0	46.0	47.2	EN 1427
Ductility at 25 °C (cm)	58.1	>100	>150	[27]
Fraass breaking point (°C)	−18	−10	−12	EN 12593
Plasticity interval (°C)	44	56	59.2	PI = SP − FBP
Penetration index	0.16	−0.65	−0.70	EN 12591
Resistance to hardening at 163 °C (RTFOT method):				EN 12607-1
mass change (%)	0.35	0.03	0.13	
softening point after RTFOT (°C)	45.6	52.2	53.0	
penetration at 25 °C after RTFOT (0.1 mm)	91	55	48	
softening point change (°C)	6.6	6.2	5.8	
residual penetration (%)	36.8	77.5	58.0	
Adhesion to gravel (mark)	2.5	2.5	3.0	[25,28]

**Table 2:** Grading of the mineral powder.

Fraction (mm)	Content (wt.%)
0.315/0.63	0.1
0.14/0.315	1.1
0.071/0.14	14.1
<0.071	84.1
Total	100.00

**Table 3:** Conditions for obtaining FMT.

Process Parameter	FMT-1	FMT-2	FMT-3	FMT-4
Raw	T1			
Formalin amount (including formaldehyde) (kg/100 kg raw)	1.0	1.9	3.0	3.2
Catalyst	H_2_SO_4_			HCl
Catalyst amount (kg/100 kg raw)	1.1	1.7	3.2	3.1
MRFC	0.91	1.12	0.94	1.0
Temperature (°C)	110	105	110	100
Duration (hours)	0.6	0.6	0.8	1.0

**Table 4:** Physical and mechanical properties of FMTs.

Index	T1	FMT-1	FMT-4	Requirements for Bitumen Brand BND 70/100 [38]	FMT-2	Requirements for Bitumen Brand BMPA 70/100-55 [39]	FMT-3	Requirements for Bitumen Brand BMPP 35/50-70 [39]
Penetration at 25 °C (0.1 mm)	247	144	131	71/100	89	71/100	47	35/50
Softening point (°C)	39.0	48.0	47.4	45–51	59.0	≥55	83.4	≥70
Ductility at 25 °C (cm)	58.1	42	59	≥60	16	≥10	4	≥ 8
Elasticity at 25 °C (%)	–	–	–	not normalized	–	≥55	–	≥75
Fraass breaking point (°C)	−18	−17	−14	≤−13	−15	−18/−14	−9	−16/−12
Penetration index	0.15	0.53	0.33	−2/1	1.14	not normalized	2.74	not normalized
Plasticity interval (°C)	57	65.0	61.4	not normalized	74.0	not normalized	92.4	not normalized
Adhesion to gravel (mark)	2.5	3.5	4.5	not normalized	4.5	≥4.5	5.0	≥4.5

**Table 5:** Composition of SMAM-15.

Name of Material	Content of Material in Asphalt Concrete (wt.%)
1 Aggregates	85
including factions	
10/15 mm	50
5/10 mm	20
0.071/5 mm	15
2 Mineral powder	15
3 Celbit	0.4
4 Bitumen (OB2, FMT-1, FMT-2, FMT-3 and FMT-4)	6.5

**Table 6:** Physical and mechanical properties of SMA-15.

Index	OB2	FMT-1	FMT-2	FMT-3	FMT-4	Requirements for SMA-15 [29]
Average density (g/cm^3^)	2.41	2.39	2.37	2.38	2.40	–
Water-saturation (vol.%)	1.5	2.0	2.3	2.5	2.1	1.0–3.0
Compression strength (MPa) at:						
20 °C	2.7	1.5	1.4	2.2	2.6	≥2.1
50 °C	1.7	0.6	0.7	1.0	1.1	≥0.6
Compression strength after water-saturation (MPa) at:						
20 °C	–	–	–	–	2.2	–
50 °C	1.8	1.3	1.7	2.3	1.2	–

## Data Availability

Data supporting reported results are stored by M. Bratychak.

## Abbreviations

Ar	arene, aromatic fragment
ARSs	asphaltene–resin substances
AT	acid tar
D25	ductility at 25 °C (cm)
FBP	Fraass breaking point (°C)
FMOB	formaldehyde-modified oxidized bitumen
FMT	formaldehyde modified tar
MRFC	mass ratio of formalin/catalyst
OB	oxidized bitumen
P25	penetration at 25 °C (0.1 mm)
PI	plasticity interval (°C)
RTFOT	rolling thin film oven test
S1	stone
SMA	stone mastic asphalt
SMAM	stone mastic asphalt mix
SP	softening point (°C)
T1	tar
E25	elasticity at 25 °C (%)

## References

[1] Porto M., Caputo P., Loise V., Eskandarsefat S., Teltayev B., Oliviero Rossi C. (2019). Bitumen and Bitumen Modification: A Review on Latest Advances. Appl. Sci..

[2] Pyshyev S., Gunka V., Grytsenko Y., Bratychak M. (2016). Polymer Modified Bitumen: Review. Chem. Chem. Technol..

[3] Wręczycki J., Demchuk Y., Bieliński D.M., Bratychak M., Gunka V., Anyszka R., Gozdek T. (2022). Bitumen Binders Modified with Sulfur/Organic Copolymers. Materials.

[4] Djimasbe R., Galiullin E.A., Varfolomeev M.A., Fakhrutdinov R.Z., Al-Muntaser A.A., Farhadian A. (2021). Experimental study of non-oxidized and oxidized bitumen obtained from heavy oil. Sci. Rep..

[5] Zhakirova N., Bakyt A., Sagindykov Z. (2020). Production of bitumen by oxidation of liquid waste oil products and determination of its properties. Mater. Today Proc..

[6] Thyrion F.C. (2021). Chapter 16 Asphalt Oxidation. Dev. Pet. Sci..

[7] Soenen H., Lu X., Laukkanen O.-V. (2016). Oxidation of bitumen: Molecular characterization and influence on rheological properties. Rheol. Acta.

[8] Pérez-Lepe A., Martınez-Boza F.J., Gallegos C., González O., Muñoz M.E., Santamarıa A. (2003). Influence of the processing conditions on the rheological behaviour of polymer-modified bitumen. Fuel.

[9] Honarmand M., Tanzadeh J., Beiranvand M. (2019). Bitumen and its modifier for use in pavement engineering. Sustainable Construction and Building Materials.

[10] Porot L., Vansteenkiste S., Makowska M., Carbonneau X., Zhu J., Damen S., Plug K. (2021). Characterisation of complex polymer modified bitumen with rheological parameters. Road Mater. Pavement Des..

[11] Alghrafy Y.M., El-Badawy S.M., Alla E.-S.M.A. (2021). Rheological and environmental evaluation of sulfur extended asphalt binders modified by high- and low-density polyethylene recycled waste. Constr. Build. Mater..

[12] Wen G., Zhang Y., Zhang Y., Sun K., Fan Y. (2002). Improved properties of SBS-modified asphalt with dynamic vulcanization. Polym. Eng. Sci..

[13] Jasso M., Hampl R., Vacin O., Bakos D., Stastna J., Zanzotto L. (2015). Rheology of conventional asphalt modified with SBS, Elvaloy and polyphosphoric acid. Fuel Process. Technol..

[14] Ortega F.J., Navarro F.J., García-Morales M. (2017). Dodecylbenzenesulfonic Acid as a Bitumen Modifier: A Novel Approach To Enhance Rheological Properties of Bitumen. Energy Fuels.

[15] Peng C., Chen P., You Z., Lv S., Zhang R., Xu F., Zhang H., Chen H. (2018). Effect of silane coupling agent on improving the adhesive properties between asphalt binder and aggregates. Constr. Build. Mater..

[16] Cuadri A., Partal P., Navarro F., García-Morales M., Gallegos C. (2011). Bitumen chemical modification by thiourea dioxide. Fuel.

[17] Gunka V., Prysiazhnyi Y., Demchuk Y., Hrynchuk Y., Sidun I., Reutskyy V., Bratychak M. (2022). Production of Bitumen Modified with Low-Molecular Organic Compounds from Petroleum Residues. 5. Use of Maleic Anhydride for Foaming Bitumens. Chem. Chem. Technol..

[18] Gunka V., Prysiazhnyi Y., Hrynchuk Y., Sidun I., Demchuk Y., Shyshchak O., Bratychak M. (2021). Production of Bitumen Modified with Low-Molecular Organic Compounds from Petroleum Residues. 2. Bitumen Modified with Maleic Anhydride. Chem. Chem. Technol..

[19] Singh B.P., Kumar L., Gupta M.M., Chauhan G. (2013). Polymer-modified bitumen of recycled LDPE and maleated bitumen. J. Appl. Polym. Sci..

[20] Moschopedis S.E., Speight J.G. (1976). Chemical Modification of Bitumen Heavy Ends and Their Non-Fuel Uses.

[21] Astakhova O., Shved M., Zubal O., Shyshchak O., Prysiazhnyi Y., Bruździak P., Bratychak M. (2019). Obtaining of Coumarone-Indene Resins Based on Light Fraction of Coal Tar. 4. Bitumen-Polymer Blends with Participation of Coumarone-Indene Resins with Epoxy Groups. Chem. Chem. Technol..

[22] Bratychak M., Astakhova O., Prysiazhnyi Y., Shved M., Shyshchak O., Namiesnik J., Plonska-Brzezinska M. (2018). Obtaining of Coumarone-Indene Resins Based on Light Fraction of Coal Tar. 3. Coumarone-Indene Resins with Methacrylic Fragments. Chem. Chem. Technol..

[23] Demchuk Y., Sidun I., Gunka V., Pyshyev S., Solodkyy S. (2018). Effect of Phenol-Cresol-Formaldehyde Resin on Adhesive and Physico-Mechanical Properties of Road Bitumen. Chem. Chem. Technol..

[24] Gunka V., Demchuk Y., Pyshyev S., Starovoit A., Lypko Y. (2018). The selection of raw materials for the production of road bitumen modified by phenol-cresol-formaldehyde resins. Pet. Coal.

[25] Gunka V., Demchuk Y., Sidun I., Miroshnichenko D., Nyakuma B.B., Pyshyev S. (2021). Application of phenol-cresol-formaldehyde resin as an adhesion promoter for bitumen and asphalt concrete. Road Mater. Pavement Des..

[26] Pyshyev S., Gunka V., Grytsenko Y., Shved M., Kochubei V. (2017). Oil and gas processing products to obtain polymers modified bitumen. Int. J. Pavement Res. Technol..

[27] (2019). Bitumen and Bituminous Binders—Determination of the Tensile Properties of Modified Bitumen by the Force Ductility Method.

[28] (2005). Viscous Road Oil Bitumen. The Method to Determine the Index of Engagement with the Surface of Glass and Rock Materials.

[29] DSTU B V. (2015). Stone Mastic Road Concrete Mix and Stone Mastic Asphalt. Specifications.

[30] (2016). Asphaltic Concrete Mixtures, Road and Aerodromes Asphaltic Concrete. Test Methods.

[31] Moshchinskaya N.K. (1969). Polimernye Materialy na Osnove Aromaticheskih Uglevodorodov i Formal’degida; Polymeric Materials Based on Aromatic Hydrocarbons and Formaldehyde.

[32] Higashihara G., Okoshi A. (2016). Aromatic Hydrocarbon Formaldehyde Resin, Modified Aromatic Hydrocarbon Formaldehyde Resin, and Epoxy Resin, and Method for Producing Said Resins. European Patent.

[33] Bratychak M., Gunka V., Prysiazhnyi Y., Hrynchuk Y., Sidun I., Demchuk Y., Shyshchak O. (2021). Production of Bitumen Modified with Low-Molecular Organic Compounds from Petroleum Residues. 1. Effect of Solvent Nature on the Properties of Petroleum Residues Modified with Folmaldehyde. Chem. Chem. Technol..

[34] Gunka V., Demchuk Y., Sidun I., Kochubei V., Shved M., Romanchuk V., Korchak B. (2020). Chemical modification of road oil bitumens by formaldehyde. Pet. Coal.

[35] Gunka V., Bilushchak H., Prysiazhnyi Y., Demchuk Y., Hrynchuk Y., Sidun I., Shyshchak O., Bratychak M. (2022). Production of Bitumen Modified with Low-Molecular Organic Compounds from Petroleum Residues. 4. Determining the Optimal Conditions for Tar Modification with Formaldehyde and Properties of the Modified Products. Chem. Chem. Technol..

[36] Ţucureanu V., Matei A., Avram A.M. (2016). FTIR Spectroscopy for Carbon Family Study. Crit. Rev. Anal. Chem..

[37] Parker F.S. (1971). Applications of Infrared Spectroscopy in Biochemistry, Biology, and Medicine.

[38] (2019). Viscous Petroleum Road Bitumens. Specification. Bitumen and bituminous binders.

[39] (2021). Polymer modified road bitumen. Specification.

